# A Study on Irritable Bowel Syndrome (IBS) in Mental Health Professionals and the Psychosocial Factors Affecting This

**DOI:** 10.1192/j.eurpsy.2024.1022

**Published:** 2024-08-27

**Authors:** J. Hong

**Affiliations:** psychiatry, Iksan hospital, iksan, Korea, Republic Of

## Abstract

**Introduction:**

he present study investigates irritable bowel syndrome in mental health professionals and the characteristics of psychosocial factors that affect this.

**Objectives:**

**Methods:**

The present study selected an irritable bowel syndrome group among 291 mental health professionals based on the Rome III criteria, and investigated demographic variables. The Hospital Anxiety Depression Scale (HADS), Psychosocial Well-being Index (PWI), and Korean Occupational Stress Scale (KOSS) were used to evaluate psychosocial factors. An independent t-test and chi-square test were used to determine differences between the groups, and a logistic regression analysis was used to determine the odds ratio (OR) of IBS based on occupational stress. SPSS 21.0 (IBM Statistical Package for the Social Sciences 21.0) was utilized for all statistics.

**Results:**

Differences in demographic variables based on IBS group were not statistically significant. Depressive symptoms (t = -4.767, p<0.001) and anxiety (t = -4.068, p<0.001) were higher in the IBS group, and psychosocial well-being was lower (t = 2.288, p<0.05). The OR of IBS based on depressive symptoms was 5.737 (95% CI = 2.24–14.69). There were significant differences in occupational stress based on IBS within the subordinate domains of physical environment (t = -3.160, p<0.01), job demand (t = -3.273, p<0.01), interpersonal conflict (t = -2.295, p<0.05), job security (t = -3.005, p<0.01), and lack of reward (t = -2.046, p<0.05). The OR of IBS based on the subordinate domains of occupational stress was 3.708 (95% CI = 1.20-11.41) in physical environment, and 3.759 (95% CI = 1.33-10.56) in job demand.

**Conclusions:**

The results of the present study verify that psychosocial factors in mental health professionals have a close correlation with IBS. Accordingly, improvements in both IBS symptoms and quality of life should occur through proactive intervention in these variables.

**Disclosure of Interest:**

None Declared

